# Supplementary material for: Risk of colorectal cancer in patients with alcoholism: A nationwide, population-based nested case-control study
Source: PLoS One. 2020 May 12;15(5):e0232740. doi: 10.1371/journal.pone.0232740 (PMC7217430; doi:10.1371/journal.pone.0232740)
Supplement: S1 Table — (DOCX) [file pone.0232740.s001.docx]

| **Table S1. Survival among CRC patients with and without alcoholism exposure** | | | |
| --- | --- | --- | --- |
| **Alcoholism exposure**  **N** | **Mortality (tracking)** | **Survival (tracking)** | ***P*** |
|  | **N (%)** | **N (%)** |  |
| With 4,197 | 418 (9.96) | 3,779 (90.04) | 0.095 |
| Without 44,898 | 4,121 (9.18) | 40,777 (90.82) |  |
| Overall 49,095 | 4,539 (9.25) | 44,556 (90.75) |  |
| **CRC=colorectal cancer, *P*: Chi-square test** | | | |
