# Supplementary material for: Risk of colorectal cancer in patients with alcoholism: A nationwide, population-based nested case-control study
Source: PLoS One. 2020 May 12;15(5):e0232740. doi: 10.1371/journal.pone.0232740 (PMC7217430; doi:10.1371/journal.pone.0232740)
Supplement: S2 Table — (DOCX) [file pone.0232740.s002.docx]

| **Table S2. Medical cost of the CRC with and without alcoholism exposure** | | | | | |
| --- | --- | --- | --- | --- | --- |
| **Medical cost (NT$)** | **N** | **mean ± SD (median)** | | ***P*** | **Scheffe post hoc** |
| **CRC** |  |  |  | <0.001 |  |
| Without | 147,285 | 53,453.12 ± 79,486.76 (28,162.00) | |  |  |
| With | 49,095 | 65,572.27 ± 99,358.80 (32,940.00) | |  |  |
| **Alcoholism exposure** |  |  |  | <0.001 |  |
| Without | 182,192 | 50,679.18 ± 78,092.20 (26,303.00) | |  |  |
| With | 14,188 | 65,434.87 ± 105,820.82 (32,605.50) | |  |  |
| **Groups** |  |  |  | <0.001 | 4 = 2 > 3 > 1 |
| 1. Without CRC, Without Alcoholism exposure | 137,294 | 44,507.22 ± 68,102.29 (23,719.00) | |  |  |
| 2. Without CRC, With Alcoholism exposure | 44,898 | 65,371.57 ± 109,522.84 (32,493.00) | |  |  |
| 3. With CRC, Without Alcoholism exposure | 9,991 | 54,104.13 ± 80,213.49 (28,501.50) | |  |  |
| 4. With CRC, With Alcoholism exposure | 4,197 | 65,591.03 ± 100,563.05 (33,011.00) | |  |  |
| ***P*: t-test for CRC and alcoholism exposure; one-way ANOVA with Scheffe post hoc for groups** | | | | | |
