# Supplementary material for: Risk of colorectal cancer in patients with alcoholism: A nationwide, population-based nested case-control study
Source: PLoS One. 2020 May 12;15(5):e0232740. doi: 10.1371/journal.pone.0232740 (PMC7217430; doi:10.1371/journal.pone.0232740)
Supplement: Attachment — Submitted filename: Responses to Reviewers.docx [file pone.0232740.s003.docx]

Yu Ru Kou, PhD

Academic Editor

PLOS ONE

PONE-D-19-23287

Risk of Colorectal Cancer in Patients with Alcoholism: A Nationwide, Population-Based Nested Case-Control Study

Dear editor Yu Ru Kou:

We have revised our manuscript and thoughtfully addressed the critiques provided by reviewers. We have highlighted that the results of this study need to be interpreted wthin the context of some limitations. In addition, we have taken into considerations of suggestions by the reviewers. Accordingly, we have presented renewed study results in our revised manuscript. We have highlighted amendments in red fonts. Specifically, our specific responses to the reviewer comments are as follows.

**Reviewer 1**

Major comments

1. Thank you for your comments. We have taken into consideration for the association between the survival of CRC patients with alcoholism exposure. The statistic results are listed below which showed that alcoholism exposure had no significant impact on the survival of CRC patients (90.04% vs. 90.82%, *P* = 0.095). However, further randomized large-scale prospective studies are needed to elucidate this issue. We have raised these points in the revised discussion part (page 15 lines 19-24).

| **Table S1. Survival among CRC patients with and without alcoholism exposure** | | | |
| --- | --- | --- | --- |
| **Alcoholism exposure**  **n** | **Mortality (tracking)** | **Survival (tracking)** | ***P*** |
|  | **n (%)** | **n (%)** |  |
| With 4,197 | 418 (9.96) | 3,779 (90.04) | 0.095 |
| Without 44,898 | 4,121 (9.18) | 40,777 (90.82) |  |
| Overall 49,095 | 4,539 (9.25) | 44,556 (90.75) |  |
| **CRC=colorectal cancer, *P*: Chi-square test** | | | |

2. We divided the patients into four groups based on with or without CRC and alcoholism exposure, and calculated the medical cost of each subgroups. The statistic results are listed below which showed that CRC patients with alcoholism exposure (Group 4) had higher medical cost than those without alcoholism exposure (Group 3) (mean, 65,591.03 and 54,104.13, *P*<0.001). We have raised these points in the revised discussion part (page 15 lines 19-24).

| **Table S2. Medical cost of the CRC with and without alcoholism exposure** | | | | | |
| --- | --- | --- | --- | --- | --- |
| **Medical cost (NT$)** | **n** | **mean ± SD (median)** | | ***P*** | **Scheffe post hoc** |
| **CRC** |  |  |  | <0.001 |  |
| Without | 147,285 | 53,453.12 ± 79,486.76 (28,162.00) | |  |  |
| With | 49,095 | 65,572.27 ± 99,358.80 (32,940.00) | |  |  |
| **Alcoholism exposure** |  |  |  | <0.001 |  |
| Without | 182,192 | 50,679.18 ± 78,092.20 (26,303.00) | |  |  |
| With | 14,188 | 65,434.87 ± 105,820.82 (32,605.50) | |  |  |
| **Groups** |  |  |  | <0.001 | 4 = 2 > 3 > 1 |
| 1. Without CRC, Without Alcoholism exposure | 137,294 | 44,507.22 ± 68,102.29 (23,719.00) | |  |  |
| 2. Without CRC, With Alcoholism exposure | 44,898 | 65,371.57 ± 109,522.84 (32,493.00) | |  |  |
| 3. With CRC, Without Alcoholism exposure | 9,991 | 54,104.13 ± 80,213.49 (28,501.50) | |  |  |
| 4. With CRC, With Alcoholism exposure | 4,197 | 65,591.03 ± 100,563.05 (33,011.00) | |  |  |
| ***P*: t-test for CRC and alcoholism exposure; one-way ANOVA with Scheffe post hoc for groups** | | | | | |

**Reviewer 2**

Major comments

1. Thank you for your comments. It is more appropriate and clear to use the term “risk of CRC” instead of “CRC” alone. (page 3 lines 6,24; page 5 line 23 and page 6 line 2).

2. Thank you for your kindly remind. The abbreviation of “OR” had been defined as “odds ratio” (page 3 line 17).

3. CRC is a multifactorial disease with complicated etiologies. The important confounders in the study, such as age, gender and comorbidities, for the association between alcoholism exposure and CRC risk had been adjusted with multivariable logistic regression analysis. Colorectal polyp is also one of the important CRC risk factor, and should be one of the confounding factor in the study. We have re-analyzed the data with adding colorectal polyp (ICD-9-CM codes 211.3-211.4, 569.0) as one of the comorbidities. However, the result still showed that alcoholism was an independent risk factor for CRC (adjusted OR, 1.631; 95% CI, 1.565–1.699). We have revised the abstract (page 3 lines 21-22), methods (page 9 lines 14-15), results (page 11 line 14,24 and page 12 lines 3-6,9-11,21-22) and discussion parts (page 13 line 2,17and page 14 lines 9-11), and add the related references (ref. 29-30).

We are enthusiastic on resubmission of our revised manuscript to your esteemed Journal for publication.

Sincerely yours,

Chao-Yang Chen, MD

Division of Colorectal Surgery, Department of Surgery

Tri-Service General Hospital, National Defense Medical Center

Taipei, Taiwan, Republic of China
