# Supplementary material for: Risk of colorectal cancer in patients with alcoholism: A nationwide, population-based nested case-control study
Source: PLoS One. 2020 May 12;15(5):e0232740. doi: 10.1371/journal.pone.0232740 (PMC7217430; doi:10.1371/journal.pone.0232740)
Supplement: Attachment — Submitted filename: Responses to reviewers.docx [file pone.0232740.s004.docx]

Yu Ru Kou, PhD

Academic Editor

PLOS ONE

PONE-D-19-23287

Risk of Colorectal Cancer in Patients with Alcoholism: A Nationwide, Population-Based Nested Case-Control Study

Dear editor Yu Ru Kou:

We have revised our manuscript and thoughtfully addressed the critiques provided by reviewers. In addition, we have taken into considerations of suggestions by the reviewers. Accordingly, we have presented renewed study results in our revised manuscript. We have highlighted amendments in red fonts. Specifically, our specific responses to the reviewer comments are as follows.

**Reviewer 1**

1. The English writing could be better.

Thanks for your kind reminder. We had sent the manuscript for English editing.

**Reviewer 3**

Major comments

1. Controls are matched to cases based on age, gender, index date, and

number of medical visits. However, on page 9, authors also mention something about matching cases and controls on residential urbanization level (6th line). I do not think this was actually done based on results presented in Table 1. Authors should clarify their statements.

Thanks for your kind reminder. It is the typing error, and we had removed the contents from the manuscript (page 9).

2. Medical events (including the diagnosis of CRC) were identified from 2000-2013. Since alcoholism and duration of alcoholism are key predictors for the models of interest, were the authors able to go back to 1995 to evaluate this exposure? Authors should clarify the time period for identifying case/control status and the time period reviewable for alcoholism.

Since alcoholism and duration of alcoholism are key predictors for the models of interest, we had re-evaluated the exposure of the time period from 1997 to 2013 (the data from 1995 to 1996 is not integrated). The result, as data showed below, still showed the time-dependent relationship between the duration of alcoholism and CRC risk (*P* for trend: 0.001). Besides, we also revised our Table 4 and the associated statements in the text (page 3 lines 19-21; page 12 lines 17-22; page 13 line 10, 23). Thanks for your opinions.

| **CRC among different retrospective duration by using multivariable logistic regression, 1997-2013** | | | |
| --- | --- | --- | --- |
| **Retrospective duration (years)** | **Alcoholism exposure** | | **With CRC vs. Without CRC** |
|  | **With CRC (N)** | **Without CRC (N)** | **Adjusted OR (95%CI)** |
| ≦0.5 | 538 | 965 | 1.501 (1.387-1.601) *** |
| >0.5, ≦1 | 679 | 1,142 | 1.530 (1.434-1.697) *** |
| >1, ≦2 | 467 | 1,203 | 1.529 (1.431-1.684) *** |
| >2, ≦3 | 397 | 972 | 1.538 (1.465- 1.701) *** |
| >3, ≦4 | 312 | 775 | 1.521 (1.412-1.672) *** |
| >4, ≦5 | 252 | 674 | 1.567 (1.488-1.724) *** |
| >5, ≦6 | 214 | 602 | 1.538 (1.467-1.701) *** |
| >6, ≦7 | 209 | 533 | 1.506 (1.398-1.635) *** |
| >7, ≦8 | 124 | 407 | 1.529 (1.432-1.684) *** |
| >8, ≦9 | 131 | 385 | 1.548 (1.472-1.708) *** |
| >9, ≦10 | 104 | 317 | 1.572 (1.497-1.735) *** |
| >10, ≦11 | 156 | 420 | 1.602 (1.524-1.779) *** |
| >11, ≦12 | 174 | 384 | 1.595 (1.501-1.768) *** |
| >12, ≦13 | 131 | 435 | 1.683 (1.533-1.811) *** |
| >13, ≦14 | 143 | 406 | 1.705 (1.597-1.852) *** |
| >14 | 166 | 371 | 1.737 (1.623-1.897) *** |
| **Mean ± SD (Median)** | 4.79 ± 5.76 (4.29) | 4.62 ± 5.23 (4.09) | ***P* for trend: 0.001** |
| **Adjusted OR = Adjusted odds ratio: Adjusted for the variables listed in Table 2.; CI = confidence interval**  **Retrospective duration: The last alcoholism exposure diagnosis before the first CRC diagnosis**  ****P* < .05, ***P* < .01, ****P* < .001** | | | |

3. I am very confused by some of the results presented in Table 2 for the

crude OR, in particular, but might carry over to the adjusted OR as well. Specifically, for age group, cases and controls were matched on age and based on Table 1, the percentages in the different age groups between cases and controls are identical. I do not see how the authors find a significant OR for age. I actually used the numbers presented in Table 1 for age group and cases and controls to run a logistic regression and find a non-significant result (p-value of 1). The crude OR for gender (though not significant, as expected, since this was a matching variable) doesn't seem to match what you would get with the numbers presented in Table 1. Similar for other crude ORs presented in Table 2. Authors should carefully check all ORs and confidence intervals and, if needed, clarify what is meant by crude OR (if it is not a simple univariate logistic regression with the listed variable as the single predictor of case status).

In Table 1, cases and controls are matched based on “percentages and distributions” of gender, age, index date, and annual medical visits. The variables mentioned above between cases and controls in Table 1 are identical; in Table 2, the listed variables under logistic regression analysis showed the “association” between the variables and CRC. We had removed the Crude OR column on Table 2 to make it clear. Thanks for your opinions.

Minor points

1. In the Statistical Analysis section, the authors have a sentence about the dose-response effect of CRC (Further analysis was performed....). This sentence either refers to analyses not presented in the results, or the authors switch CRC and alcoholism. I believe the authors mean to say a dose-response effect of alcoholism on risk of CRC. They further state based on the "average number of medical visits for CRC", but the presented results are based on duration in years of alcoholism, which even if CRC is replaced by alcoholism, doesn't necessarily correspond to number of medical visits. Authors should clarify this statement.

Thanks for your kind reminder. The statement should be revised as “Further analysis was performed for assessing the dose-response effect of alcoholism on risk of CRC according to the duration in years of alcoholism” (page 10 lines 5-7).

2. On page 11, 2nd paragraph, sentence starting with "In addition, patients in

the case group..", "were significantly higher in the case group" should be changed to "than the control group".

Thanks for your kind reminder. The statement should be revised as “In addition, patients in the case group were more likely to have comorbidities. COPD, DM, CAD, HTN, peptic ulcer, IBD, and colorectal polyp than the control group (P < .05)” (page 11 lines 12-14)

3. The note under Table 2 does not list all of the variables included in the adjusted model, since not all variables included in the model are included in the table.

We had revised the description note under Table 2 as “Adjusted for the variables listed in Table 1” to make it clear. Thanks for your suggestion.

4. Table 4: authors should include information about how many cases/controls had alcoholism by the different durations. Also, in these models was the reference group no alcoholism, so that, for example, in the >5 years results, those with alcoholism for less than 5 years were excluded from the analysis? Also, the note about retrospective duration is confusing, so should be clarified.

Thanks for your kind reminder. We had revised the Table 4 to include the information about the numbers of cases/controls had alcoholism. Besides, in the >5 years results means those with alcoholism for less than 5 years were excluded from the analysis.

5. Page 13, 3rd line "is the first large population analysis shows" should be "is the first large population-based analysis that shows"

It is indeed more appropriate to revise the statement as “this study is the first large population-based analysis that shows a possible direct correlation between CRC and alcoholism (page13 lines 3-4).

6. Authors provide supplemental tables but do not refer to them in the text anywhere.

Thanks for your kind reminder. We had included the supplemental tables and referred them in the text (page 15 line 23).

We are enthusiastic on resubmission of our revised manuscript to your esteemed Journal for publication.

Sincerely yours,

Chao-Yang Chen, MD

Division of Colorectal Surgery, Department of Surgery

Tri-Service General Hospital, National Defense Medical Center

Taipei, Taiwan, Republic of China
