# Supplementary material for: Risk of colorectal cancer in patients with alcoholism: A nationwide, population-based nested case-control study
Source: PLoS One. 2020 May 12;15(5):e0232740. doi: 10.1371/journal.pone.0232740 (PMC7217430; doi:10.1371/journal.pone.0232740)
Supplement: Attachment — Submitted filename: Responses to reviewers.docx [file pone.0232740.s005.docx]

Yu Ru Kou, PhD

Academic Editor

PLOS ONE

PONE-D-19-23287

Risk of Colorectal Cancer in Patients with Alcoholism: A Nationwide, Population-Based Nested Case-Control Study

Dear editor Yu Ru Kou:

We have revised our manuscript and thoughtfully addressed the critiques provided by reviewers. In addition, we have taken into considerations of suggestions by the reviewers. We have highlighted amendments in red fonts. Specifically, our specific responses to the reviewer comments are as follows.

**Reviewer 3**

Minor points

1. On page 11, the authors added a sentence "In addition...". The previous statement already states that there are differences between groups in comorbidities, so the authors may want to use "In particular" instead. They also end the sentence after "comorbidities" but that makes the next sentence an incomplete sentence.

Thanks for your kind reminder. We have revised our sentence (page 11 lines 12-14).

2. On page 11, the authors state that most CRC patients were treated in Northern and Southern Taiwan - according to Table 1, Middle Taiwan should also be stated, since a slightly higher percentage of CRC patients came from there than from Southern Taiwan.

Thanks for your kind reminder. We have revised our statement (page 11 line 15).

3. On page 11, "an combination" should be "a combination"

Thanks for your kind reminder. We have revised our typing error (page 11 line 16).

4. On page 11, last paragraph, the authors mention that Table 2 includes univariate results. The authors opted to remove these results from the table based on my earlier critique (I actually think they are useful, though the authors would then need to check results, because their explanation in their response for the age difference that I questioned is not adequate). I leave it to the editor to decide whether the univariate results need to be presented. If they are not, authors should not refer to them in the text.

We have revised our statement in the text. Thanks for your suggestions (page 11 lines 18-19).

5. On page 12, the authors mention findings from stratified analyses based on urbanization levels and healthcare levels without giving relevant statistics or data. Authors are encouraged to either include OR and 95% CI in the text to support the statement, add the results to Table 3 or add a supplemental table that includes the information.

We have revised the text of stratified analyses based on urbanization levels and healthcare levels with OR and 95% CI to support our statement (page12 lines 11-18). We also have added relevant statistics and data in table 3. Thanks for your suggestions.

We are enthusiastic on resubmission of our revised manuscript to your esteemed Journal for publication.

Sincerely yours,

Chao-Yang Chen, MD

Division of Colorectal Surgery, Department of Surgery

Tri-Service General Hospital, National Defense Medical Center

Taipei, Taiwan, Republic of China
